# Caesarean section use and inequalities in Bangladesh in regional context: comparisons with West Bengal and Nepal

**DOI:** 10.1080/16549716.2026.2750044

**Published:** 2026-10-22

**Authors:** Shaki Aktar, Monjur Rahman

**Affiliations:** a Maternal and Child Health Division, International Center for Diarrhoeal Disease Research, Bangladesh (ICDDR,B), Dhaka, Bangladesh; b Rural Health Research Institute, Charles Sturt University, Orange, NSW, Australia

**Keywords:** C-section, private healthcare, health equity, South Asia, inequality, concentration Index

## Abstract

**Background:**

Caesarean section (CS) can be life-saving when medically indicated, but its rapid increase raises concerns about overuse and unequal access.

**Objective:**

To characterise CS prevalence, facility patterns, socioeconomic inequality, and associated factors in Bangladesh, and descriptively compare these patterns with Nepal and West Bengal, India.

**Methods:**

We analysed cross-sectional data from the 2022 Bangladesh and Nepal Demographic and Health Surveys and the West Bengal sample of India’s National Family Health Survey-5 (2019–2021). Analyses included each woman’s most recent live birth in the preceding 5 years. We estimated weighted prevalence, concentration indices, and adjusted prevalence ratios (APRs) with 95% confidence intervals (CIs) using survey-weighted modified Poisson regression.

**Results:**

CS prevalence was highest in Bangladesh (44.6%), followed by West Bengal (34.8%) and Nepal (19.3%). Among facility births, the proportions delivered by CS were 69.3%, 37.4%, and 23.9%, respectively. Private for-profit facility delivery showed the strongest association with CS use in Bangladesh (APR 5.86; 95% CI 5.26–6.54), West Bengal (APR 3.02), and Nepal (APR 2.65). CS use was concentrated among wealthier women, with concentration indices of 0.20, 0.24, and 0.35, respectively. In Bangladesh, CS use was also associated with urban residence and more antenatal care visits; maternal age showed stronger associations in West Bengal and Nepal.

**Conclusions:**

Bangladesh had higher CS use than West Bengal and Nepal, alongside high private-facility CS rates and substantial wealth-related inequality. Policies should strengthen monitoring and clinical audit to identify and review potentially unnecessary CS while ensuring timely access to medically indicated CS for disadvantaged women.

## Paper Context

**Main findings**: Caesarean section prevalence was highest in Bangladesh (44.6%), with particularly high CS use in private for-profit facilities and a pro-rich socioeconomic distribution, compared with West Bengal and Nepal.

**Added knowledge**: Using comparable recent population-representative survey data across Bangladesh, West Bengal, and Nepal, this study demonstrates substantial cross-setting differences in CS use and identifies private for-profit facility delivery as the strongest factor associated with CS use across all three settings.

**Global health impact for policy and action**: The findings support a dual policy approach that strengthens monitoring and clinical review of high CS use at the facility level, while ensuring equitable and timely access to medically indicated CS for disadvantaged women.

## Background

Access to comprehensive emergency obstetric care, particularly caesarean section (CS), is key to reducing maternal and neonatal mortality and to achieving Sustainable Development Goal 3 (SDG3) [1–4]. CS is a life-saving surgical intervention when life-threatening complications arise during childbirth. However, despite being widely regarded as a safe procedure, CS may carry greater risks than vaginal delivery, particularly when performed without a clear medical indication. Evidence indicates that it is associated with immediate maternal and perinatal complications, and may also have implications for future pregnancies and long-term health [5–8]. While there is no consensus on a single optimal CS rate, earlier WHO expert consultations suggested an ‘ideal’ population rate of around 10–15% [9]. In 2015, WHO updated its position, emphasizing that the priority should be ensuring access to CS for women who need it, rather than attempting to achieve a specific target rate [10]. Nevertheless, CS use has increased substantially worldwide, particularly in low- and middle-income countries (LMICs) [11]. Evidence from multiple studies, including a WHO multi-country assessment, indicates that millions of CS procedures may be unnecessary and associated with substantial costs [12,13]. As a consequence, out-of-pocket payments are increasing considerably and may expose households to catastrophic health expenditures and push them into poverty [14,15].

In the last decade, health systems in many developing countries have encouraged increased engagement of private stakeholders and public–private partnerships [16]. While private-sector expansion may contribute to improvements in antenatal care (ANC) utilisation and institutional delivery [17–19], concerns remain regarding variable quality of care and potential financial incentives that may influence surgical delivery over vaginal birth [20,21]. A large body of literature has examined types of facility utilisation and modes of delivery for childbirth in LMICs. Evidence from observational studies indicates that high CS prevalence in South Asian countries is partly driven by deliveries in private facilities, where CS use is more common than in public or not-for-profit settings [22,23]. The literature on facility choice for childbirth further indicates that variations in CS prevalence are also influenced by a range of factors. These include provider-level financial incentives and maternal characteristics, including higher socioeconomic status and educational attainment, both of which are associated with a greater likelihood of CS delivery [24–26].

Previous studies have provided important comparative evidence on CS use in Bangladesh and neighbouring settings. These studies have examined trends and determinants of CS [22,27], socioeconomic inequalities in CS use [24,25], and utilisation of private facilities for childbirth [26], while a study comparing public and private facilities in Bangladesh, India, and Nepal was based on selected underserved communities rather than nationally representative populations [28]. Meanwhile, national CS prevalence in Bangladesh increased from 7.5% in 2007 to 14.1% in 2011, 24.3% in 2014, 32.8% in 2017–18, and 45% in 2022 [22,29]. Thus, the epidemiological context in which earlier evidence was generated may differ markedly from the current situation. Differences in study populations, geographic coverage, and study designs across previous studies may also limit the comparability of their findings and their relevance for drawing policy implications across settings. These gaps highlight the importance of comparing recent population-based evidence from settings with broadly comparable geographic, socioeconomic, and cultural characteristics.

Bangladesh, Nepal, and West Bengal, India, provide a useful comparative framework for examining CS use in South Asia because they share important historical, cultural, and demographic features while differing in socioeconomic conditions, health-system organisation, and the roles of public and private providers in childbirth care [28]. West Bengal is particularly relevant to Bangladesh because of its geographic contiguity, shared historical and cultural ties, and broadly comparable health, demographic and socioeconomic characteristics [30]. Examining West Bengal separately from India as a whole is important because national-level estimates may mask substantial subnational variations in maternal health service utilisation, particularly in a country as geographically and socioeconomically diverse as India [31]. Nepal provides a culturally and geographically proximate neighboring-country comparator to Bangladesh, while offering a distinct health-system context and lower CS use [32]. Comparing these three settings therefore enables assessment of whether the observed patterns reflect a broader South Asian phenomenon or a more pronounced Bangladesh-specific pattern.

The availability of recent nationally representative Demographic and Health Surveys (DHS) and a similarly designed National Family and Health Survey (NFHS) from all three settings provides a unique opportunity for such a comparison. These surveys use standardized methods and contain comparable information on maternal characteristics, antenatal care, and delivery care across settings [33]. By leveraging these survey data, we estimated CS prevalence and socioeconomic inequalities and examined associations of CS use with maternal characteristics and place of delivery across the three selected South Asian settings.

## Methods

### Study design and population

We conducted a secondary analysis of the most recent publicly available, population-based cross-sectional data from the Bangladesh Demographic and Health Survey (BDHS 2022), Nepal Demographic and Health Survey (NDHS 2022), and India’s National Family Health Survey-5 (NFHS-5, 2019–2021) [29,32,34]. To ensure geographic, socioeconomic, and cultural comparability with Bangladesh and Nepal, the analysis for India was restricted to the state of West Bengal.

Both the DHS and NFHS employ a stratified, multistage cluster sampling design to produce nationally representative estimates. For simplicity, both survey programmes are collectively referred to as DHS throughout this paper. From the DHS data repositories, the Births Recode datasets were extracted and harmonized. The Births Recode files contain information on pregnancies and live births reported by women of reproductive age, including pregnancy histories, birth outcomes, and maternal and delivery-related characteristics. To minimize recall bias, improve comparability of delivery-related information, avoid correlated observations from multiple births to the same woman, and ensure equal contribution of observations across respondents, the analysis was restricted to each woman’s most recent live birth within the 5 years preceding the survey. Accordingly, the analytical dataset included one observation per woman, corresponding to her most recent live birth during this period, and CS status referred specifically to the mode of delivery of that birth. The dataset comprised 5,064 women from Bangladesh, 4,894 women from West Bengal, and 2,942 women from Nepal, yielding 12,900 distinct women. No additional inclusion or exclusion criteria were applied, and no further observations were excluded because of missing information on the exposure and outcome variables.

### Outcome variable

The primary outcome was the DHS-recorded mode of delivery of the included live birth in the analytical dataset. CS delivery was operationalized as a binary variable, coded as 1 for ‘Yes’ (caesarean section) and 0 for ‘No’ (non-caesarean delivery). The ‘No’ category was used as the reference category. The analysis did not distinguish between elective and emergency caesarean sections because this information was not available in a comparable form across all three settings.

### Explanatory variables

Explanatory variables were selected a priori based on previous literature and data availability across surveys. Maternal age at birth was categorised as <21, 21–24, 25–29, and ≥30 years. Parity, defined as total children ever born, was categorised as 1, 2, 3, and ≥4 births. Maternal education was classified by years of schooling (0, 1–5, 6–10, and ≥11 years). Household socioeconomic status was measured using wealth index quintiles (poorest to richest). Place of residence was categorised as urban or rural. Child sex was a dichotomous variable indicating male or female.

Pregnancy intention was classified as wanted then, wanted later, or wanted no more. ANC utilisation was measured by the number of ANC visits and categorised as 0, 1, 2, 3, and ≥4 visits. Exposure to private-sector ANC was defined as receipt of at least one ANC visit from a private provider. Place of delivery was classified as home, public not-for-profit facilities, and private for-profit facilities. Facility birth was defined as a birth occurring in either public not-for-profit or private for-profit facilities, whereas home births were classified separately.

### Statistical analysis

All analyses accounted for the complex survey design by applying sampling weights and accounting for stratification and clustering. Sampling weights were rescaled by dividing them by 1,000,000, as recommended in the guideline, and were subsequently normalised within each study setting [35]. Because primary sampling unit identifiers can repeat across survey settings, the survey setting was combined with the cluster identifier to define survey-specific clusters for cluster-robust standard-error estimation. The data were analysed using both descriptive and analytical methods. Descriptive statistics were summarised using unweighted frequencies and weighted percentages. Differences in maternal and household characteristics across settings were assessed using chi-square tests.

Socioeconomic inequality in CS utilisation was assessed using concentration curves and the concentration index [36]. The concentration index was calculated as twice the covariance between CS use and the fractional rank of household wealth, divided by the mean of CS use. Concentration curves plotted the cumulative proportion of CS deliveries against the cumulative proportion of births ranked by household wealth. Positive concentration index values indicated a higher concentration of CS deliveries among wealthier households.

Multivariable modified Poisson regressions with a log link were used to estimate prevalence ratios, accounting for clustering within DHS primary sampling units. Separate models were fitted for Bangladesh, West Bengal, and Nepal to identify setting-specific factors associated with CS use. Multivariable models included delivery in a private for-profit facility as the primary exposure and adjusted for maternal age, parity, maternal education, household wealth quintile, place of residence, pregnancy intention, antenatal care utilisation, receipt of antenatal care from private providers, and child sex. For the primary analysis, delivery in a private for-profit facility was coded as a binary exposure, with all other places of delivery comprising the non-private reference category. This population-level analysis included all women with a recent live birth. As a sensitivity analysis, we restricted the analysis to facility births and compared private for-profit with public not-for-profit facilities using the same covariates. Results are presented as adjusted prevalence ratios (APR) with 95% confidence intervals (CI). Statistical analyses were conducted using the statsmodels package (version 0.14.0) in Python (version 3.14).

## Results

### Sample characteristics

The final pooled analytical dataset included 12,900 women and their most recent live births across the three DHS settings: 5,064 births from Bangladesh (DHS-8), 4,894 births from West Bengal, India (NFHS-5), and 2,942 births from Nepal (DHS-8). In Bangladesh, 25.5% of mothers were aged 30 years or older at the time of birth, and 37.8% were first-time mothers (Table 1). Regarding education and socioeconomic status, 16.9% of Bangladeshi mothers had 11 or more years of schooling, and 18.3% were in the richest wealth quintile. Healthcare utilisation in Bangladesh showed that 39.5% of women attended four or more ANC visits, and 27.5% received ANC from private providers. In comparison, the proportion of women with four or more ANC visits was higher in West Bengal (73.7%) and Nepal (81.4%).

**Table 1.** Distribution of maternal and household characteristics among women with a live birth in the 5 years preceding the survey: a comparative profile of Bangladesh, West Bengal (India), and Nepal.

	Total *N* = 12,900	Bangladesh *N* = 5,064	West Bengal *N* = 4,894	Nepal *N* = 2,942
	n (%)	n (%)	n (%)	n (%)
Maternal Age (years)				
<21	2,298 (16.9)	1,044 (21.4)	740 (15.4)	514 (16.0)
21–24	3,741 (30.2)	1,276 (25.6)	1,565 (32.0)	900 (29.6)
25–29	3,941 (31.9)	1,430 (27.5)	1,632 (33.7)	879 (31.1)
≥30	2,920 (21.0)	1,314 (25.5)	957 (18.8)	649 (23.3)
Parity				
1	5,386 (44.5)	1,894 (37.8)	2,306 (47.8)	1,186 (40.4)
2	4,576 (36.2)	1,761 (34.0)	1,817 (37.2)	998 (34.9)
3	1,838 (12.3)	926 (18.7)	490 (9.5)	422 (14.4)
≥4	1,100 (7.0)	483 (9.5)	281 (5.4)	336 (10.3)
Education (years)				
0	1,397 (10.0)	278 (5.6)	546 (9.6)	573 (20.1)
1–5	2,429 (17.5)	1,175 (22.8)	773 (15.7)	481 (16.2)
6–10	6,403 (51.0)	2,687 (54.7)	2,458 (51.5)	1,258 (42.0)
≥11	2,671 (21.5)	924 (16.9)	1,117 (23.2)	630 (21.7)
Wealth Status				
Poorest	3,842 (29.9)	1,053 (20.4)	1,813 (34.9)	976 (22.9)
Poor	3,077 (26.1)	1,021 (20.8)	1,428 (29.1)	628 (21.2)
Middle	2,455 (19.7)	1,008 (20.6)	901 (19.2)	546 (20.7)
Rich	1,999 (14.7)	997 (19.9)	526 (11.7)	476 (19.4)
Richest	1,527 (9.6)	985 (18.3)	226 (5.1)	316 (15.8)
Number of ANC				
0	1,077 (9.8)	410 (8.0)	584 (11.9)	83 (2.9)
1	955 (5.7)	733 (14.7)	149 (3.0)	73 (2.3)
2	1,300 (7.7)	952 (19.4)	192 (3.8)	156 (5.7)
3	1,533 (10.2)	910 (18.3)	403 (7.6)	220 (7.8)
≥4	8,035 (66.7)	2,059 (39.5)	3,566 (73.7)	2,410 (81.4)
Planned Pregnancy				
Yes	10,764 (86.5)	4,077 (80.8)	4,382 (90.2)	2,305 (79.2)
Later	1,346 (8.2)	617 (11.9)	283 (5.5)	446 (14.8)
Never	790 (5.2)	370 (7.3)	229 (4.3)	191 (6.0)
Residence Area				
Rural	8,566 (68.1)	3,396 (73.2)	3,725 (73.1)	1,445 (34.4)
Urban	4,334 (31.9)	1,668 (26.8)	1,169 (26.9)	1,497 (65.6)
Private Facility ANC				
No	9,626 (75.6)	3,732 (72.5)	3,848 (79.3)	2,046 (63.1)
Yes	3,274 (24.4)	1,332 (27.5)	1,046 (20.7)	896 (36.9)
Child Sex				
Male	6,695 (51.7)	2,603 (51.3)	2,542 (51.8)	1,550 (51.8)
Female	6,205 (48.3)	2,461 (48.7)	2,352 (48.2)	1,392 (48.2)

Notes: All values are presented as unweighted frequencies (n) and weighted percentages (%) using survey sampling weights. Data are derived from the 2022 Bangladesh Demographic and Health Survey (BDHS), 2022 Nepal Demographic and Health Survey (NDHS), and the India National Family Health Survey (NFHS-5; 2019–2021), with the Indian analysis restricted to West Bengal. Abbreviations: ANC, Antenatal Care.

### Institutional delivery and CS prevalence

Institutional delivery in Bangladesh was 64.3% (Figure 1(A)). Of these, 45.1% occurred in private facilities and 19.2% in public facilities. The overall prevalence of CS in Bangladesh was high at 44.6% (Figure 1(B)). This prevalence varied substantially by place of delivery: 34.6% of births in public facilities were by CS and 84.1% in private for-profit facilities (Figure 1(C)). Comparatively, the overall CS prevalence was lower in Nepal (19.3%) and West Bengal (34.8%). Private-sector CS rates in West Bengal (83.5%) were similar to Bangladesh, while Nepal’s had a considerably lower private-sector CS rate (46.9%).

**Figure 1.**
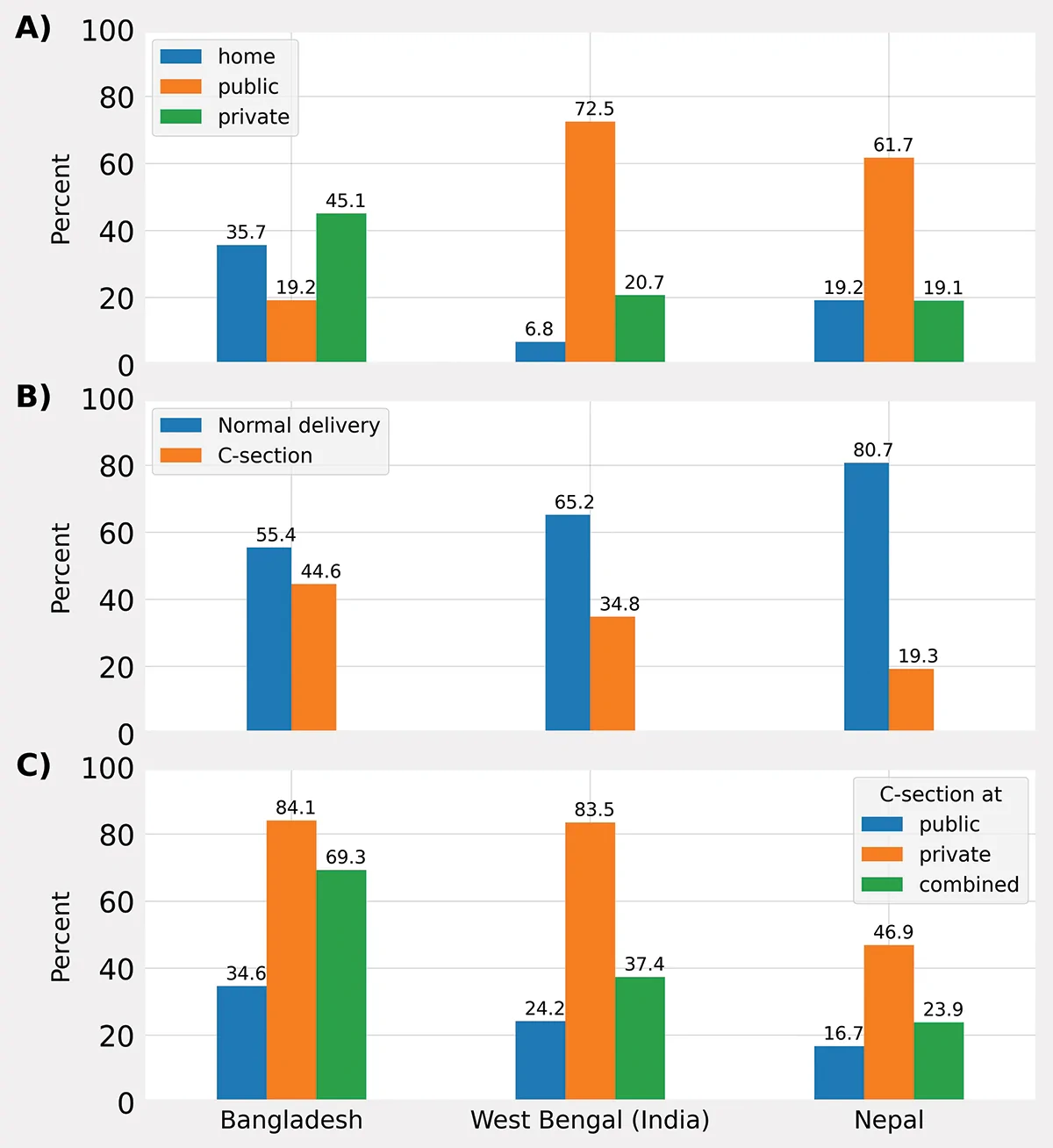
Patterns of place of delivery and caesarean section prevalence by study setting and facility type.

Note: The total number of women included in the analysis was 5,064 in Bangladesh, 4,894 in West Bengal (India), and 2,942 in Nepal, each contributing her most recent live birth. All percentages are presented as unweighted frequencies (n) and weighted percentages (%) to account for the complex survey design.

(A) Distribution of births by place of delivery. The observed numbers for home, public not-for-profit, and private for-profit facilities were 1,798, 987, and 2,279 in Bangladesh (*N* = 5,064); 362, 3,575, and 957 in West Bengal (*N* = 4,894); and 583, 1,886, and 473 in Nepal (*N* = 2,942), respectively.

(B) Overall CS prevalence. The observed numbers of CS events were 2,285 in Bangladesh (*N* = 5,064), 1,591 in West Bengal (*N* = 4,894), and 454 in Nepal (*N* = 2,942).

(C) CS prevalence among public not-for-profit facilities, private for-profit facilities, and public and private facilities combined (all facility births). The observed numbers of CS events (n/N) were 358/987, 1,927/2,279, and 2,285/3,266 in Bangladesh; 794/3,575, 797/957, and 1,591/4,532 in West Bengal (India); and 257/1,886, 197/473, and 454/2,359 in Nepal, respectively.

### Socioeconomic inequities in CS use

Concentration curves show a pro-rich inequality in Bangladesh, with a concentration index of 0.20 for CS use (Figure 2), reflecting higher CS use among wealthier households. Comparatively, West Bengal has a higher index of 0.24, while Nepal has the highest index of 0.35.

**Figure 2.**
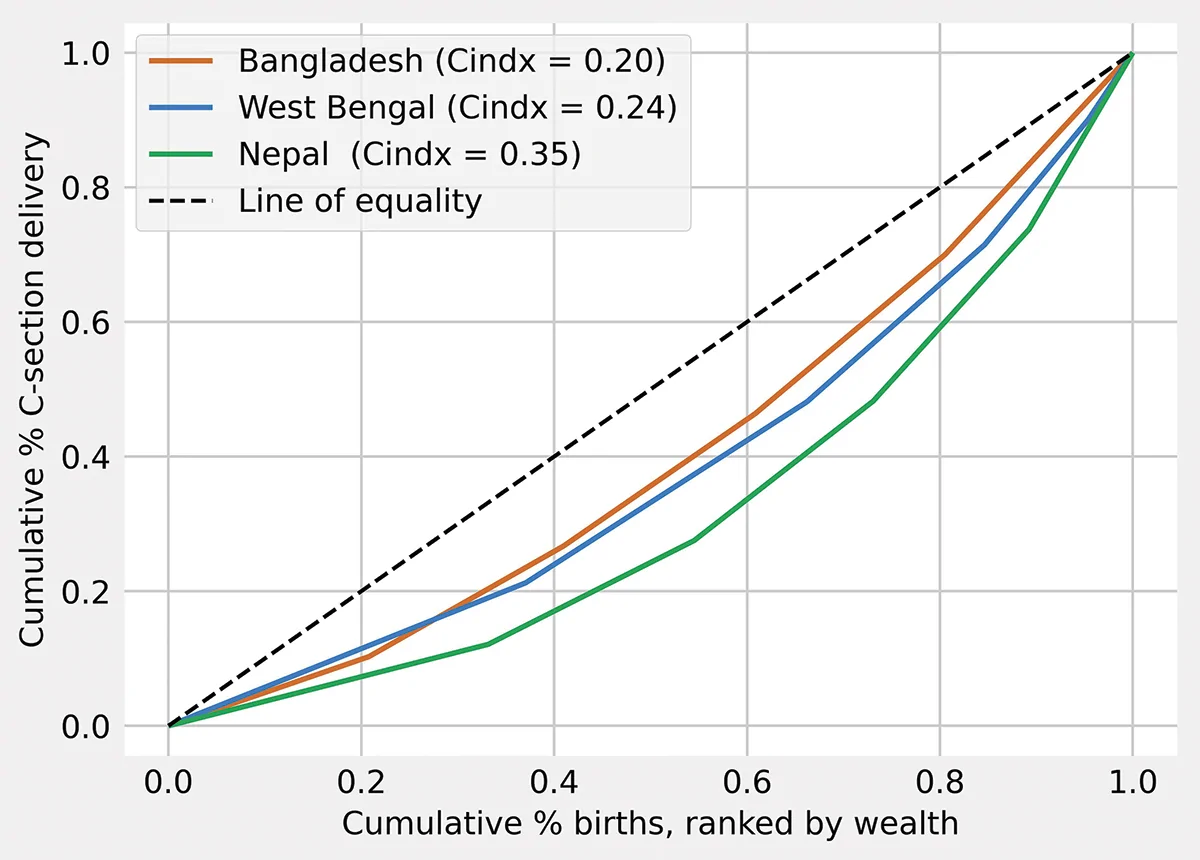
Concentration curves for caesarean section use by household wealth in Bangladesh, West Bengal (India), and Nepal.

Note: The diagonal line represents the line of perfect equality. Higher values of the concentration index (Cindx) indicate greater inequality. Curves lying below the diagonal indicate pro-rich inequality, where CS use is concentrated among wealthier households.

### Multivariable analysis of factors associated with CS use

In multivariable modified Poisson regression models, delivery in a private for-profit facility had the strongest association with CS use across all settings (Figure 3). Bangladeshi women delivering in private for-profit facilities had a substantially higher prevalence of CS (APR 5.86; 95% CI: 5.26–6.54) than women delivering outside the private facilities. The corresponding associations were substantially smaller in West Bengal (APR 3.02, 95% CI 2.79–3.26) and Nepal (APR 2.65, 95% CI 2.26–3.11). In a sensitivity analysis restricted to facility births, the association between private facility delivery and CS remained positive in all three settings, although the magnitude was lower than in the primary population-level analysis. The APRs for private for-profit versus public not-for-profit facility delivery were 2.37 (95% CI 2.17–2.58) in Bangladesh, 2.88 (95% CI 2.67–3.10) in West Bengal, and 2.29 (95% CI 1.97–2.66) in Nepal (Supplementary Figure S1).

**Figure 3.**
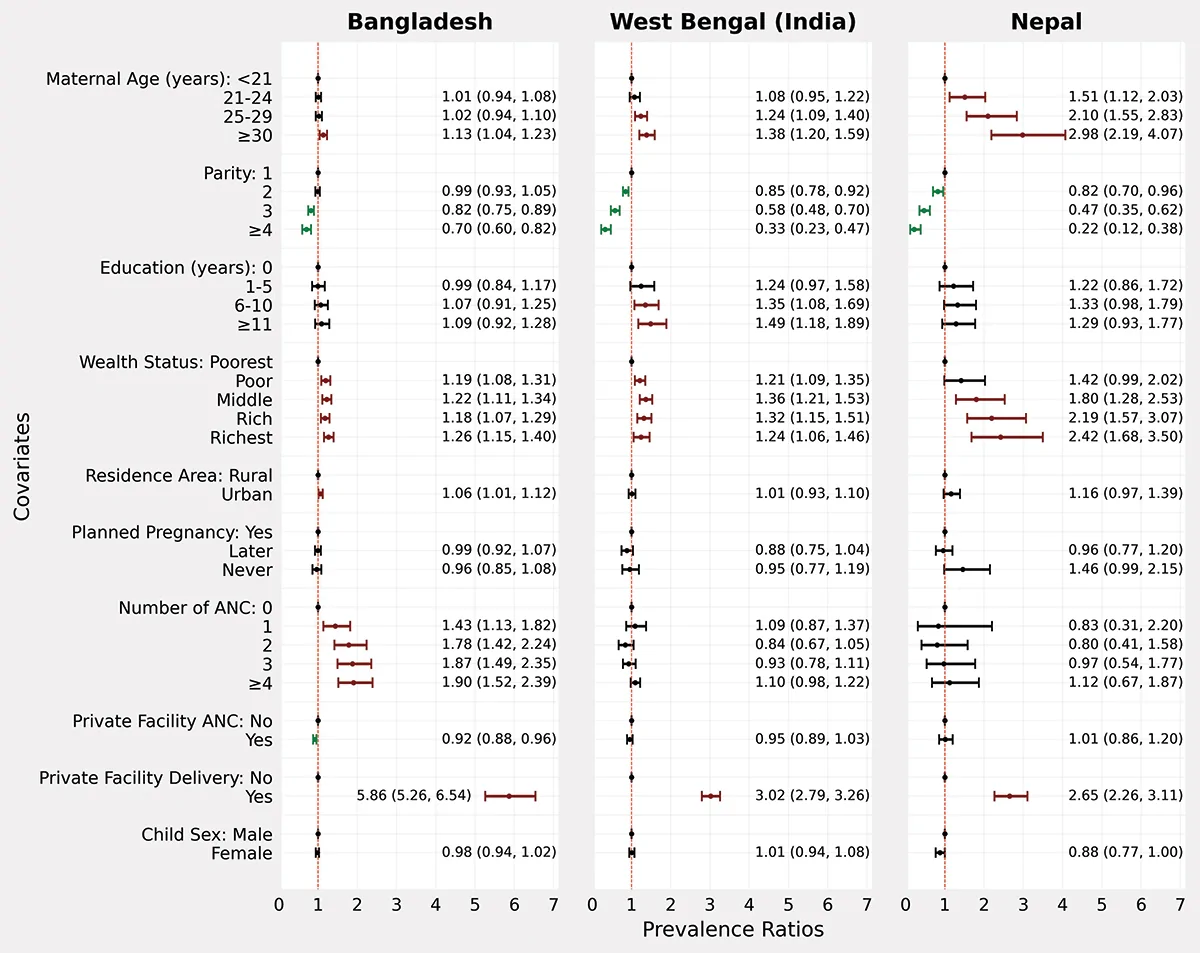
Adjusted prevalence ratios (APRs) of caesarean section delivery associated with maternal, socioeconomic, and healthcare-related factors in Bangladesh, West Bengal (India), and Nepal.

Note: Forest plots display APRs and 95% CIs derived from multivariable modified Poisson regression models with robust variance. The numbers of observations contributing to the primary models were 5,064 in Bangladesh, 4,894 in West Bengal (India), and 2,942 in Nepal. All models account for the complex DHS survey design through the application of sampling weights, and are adjusted for all displayed covariates, including maternal age, parity, education, household wealth, place of residence, pregnancy intention, and antenatal care visits.

Maternal age showed a weak association in Bangladesh, where women aged 30 or older had a slightly higher prevalence of CS (APR 1.13; 95% CI 1.04–1.23) compared with those under 21 years. In contrast, stronger positive associations between maternal age and CS were observed in West Bengal (≥30 years: APR 1.38) and Nepal (≥30 years: APR 2.98). Higher parity was associated with a lower prevalence of CS in Bangladesh, as women with four or more births had a lower prevalence (APR 0.70) than first-time mothers. This inverse association was even more pronounced in West Bengal (APR 0.33) and Nepal (APR 0.22). Child sex was not significantly associated with CS use.

Regarding socioeconomic and healthcare factors, CS use increased with household wealth in Bangladesh, where women in the richest quintile had a higher prevalence of CS (APR 1.26; 95% CI: 1.15–1.40) compared with those in the poorest. Similar wealth-related gradients were observed in West Bengal and Nepal, though the magnitude of the association was more pronounced in the latter. Maternal education was not associated with CS use in Bangladesh and Nepal, whereas higher levels of maternal education were associated with CS use in West Bengal. Urban residence showed a small positive association in Bangladesh (APR 1.06; 95% CI: 1.01–1.12), a factor that was less influential in the other two settings. Women attending four or more ANC visits had a significantly higher prevalence of CS in Bangladesh (APR 1.90; 95% CI: 1.52–2.39), but not in the other two settings. Receipt of ANC from private providers was associated with slightly lower CS use in Bangladesh (APR 0.92; 95% CI: 0.88–0.96). While pregnancy intention did not show a consistent association with CS in Bangladesh and West Bengal, unplanned pregnancies in Nepal were associated with a higher prevalence of surgical delivery, although the CI included unity (APR 1.46, 95% CI 0.99–2.15).

## Discussion

This study shows that CS is a dominant mode of delivery in Bangladesh. The overall CS prevalence was highest in Bangladesh (44.6%), compared with West Bengal, India (34.8%) and Nepal (19.3%), and the difference was particularly pronounced by facility type: 84.1% of births in private for-profit facilities in Bangladesh were delivered by CS, compared with 34.6% in public not-for-profit facilities. In multivariable analyses, delivery in a private for-profit facility was associated with the highest adjusted prevalence of CS across all three settings, with the strongest association observed in Bangladesh. CS use was concentrated among wealthier women across settings, as reflected by the positive concentration indices. These findings highlight substantial cross-setting and within-setting similarities and differences in CS use and point to the importance of facility type and socioeconomic position in understanding patterns of CS use in Bangladesh and other selected South Asian settings.

The high level of CS use observed in Bangladesh should be interpreted as a population-level pattern of utilisation rather than evidence of the clinical appropriateness or inappropriateness of individual procedures. WHO has emphasised that countries should focus on ensuring access to CS for women who need it rather than attempting to achieve a specific population-level rate [10]. The present study cannot distinguish medically indicated from potentially unnecessary CS because the DHS data do not provide sufficiently detailed information on clinical indications, urgency of delivery, previous CS, maternal request, or referral pathways [37–39]. Similarly, the lower CS prevalence observed in Nepal cannot be interpreted as evidence of appropriate use or underuse. However, the differences in population-level CS use highlight the need to further explore why CS use varies across geographically contiguous settings, even when they share similar cultural and sociodemographic characteristics [28,30].

The strongest factor associated with CS use across all settings was delivery in a private for-profit facility. The magnitude of the association was particularly large in Bangladesh. In the primary analysis, this reflects the distribution of CS use across the full population of births in relation to place of delivery. The sensitivity analysis restricted to facility births showed a similar positive association, although the effect estimates comparing private for-profit with public not-for-profit facilities were smaller. The strong association between private-facility delivery and CS is consistent with earlier South Asian evidence showing substantially higher CS use in private facilities than in public-sector settings [22,28]. However, this association should not be interpreted as a causal effect of private facility delivery, because place of delivery can be influenced by complications, referral patterns, provider advice, household preference, and financial capacity, which could not be analysed because of data limitations [16,39,40]. The very high CS rates in private facilities and the consistent associations observed across analyses indicate that the private sector is central to understanding current CS use in these South Asian settings.

Bangladesh-specific evidence provides further context for the strong association between private-sector delivery and CS. A population-based study in rural Matlab showed that only a small proportion of CS deliveries in for-profit private facilities were performed for absolute maternal indications [39]. A study using the Robson 10-group classification system in urban private hospitals reported an overall CS rate of 82%, with preterm birth, previous CS and elective CS making major contributions [38]. Qualitative studies further suggest that inadequate labour monitoring, staffing constraints, provider preference, and women’s limited involvement in decision-making may shape the final mode of delivery [37,41,42]. Together, these studies provide possible explanations for the observed association in Bangladesh, although the specific mechanisms could not be assessed using the DHS data.

The association between ANC and CS also requires careful interpretation. Higher ANC utilisation was associated with greater CS use in Bangladesh, whereas this association was absent in West Bengal and Nepal. This observational association should not be interpreted as a causal effect of ANC on CS use. Women with higher-risk pregnancies may require closer antenatal monitoring and may also have a higher likelihood of CS [39]. Higher ANC exposure may also increase contact with healthcare providers and clinic or hospital staff, providing more opportunities for provider recommendations and facility-based counselling regarding mode of delivery, although whether and how this influences CS use remains unclear. Previous CS is another important clinical factor associated with subsequent CS and may therefore confound the observed association between ANC utilisation and CS [39,43]. Other maternal, socioeconomic, and healthcare-related factors may also contribute to the observed association between ANC utilisation and CS delivery [40,44].

The association between private-sector ANC and CS was sensitive to adjustment for place of delivery. After adjustment for maternal, socioeconomic, and pregnancy-related characteristics, private-sector ANC was not clearly associated with CS in Bangladesh (APR 1.03, 95% CI 0.97–1.10) but was positively associated in West Bengal (APR 1.29, 95% CI 1.20–1.40) and Nepal (APR 1.47, 95% CI 1.26–1.71). These associations were substantially attenuated after further adjustment for private-facility delivery, becoming close to the null in West Bengal and Nepal and slightly inverse in Bangladesh. This pattern suggests that the association between ANC source and CS is closely related to subsequent place of delivery, as women receiving private ANC may be more likely to deliver in private facilities where CS use is higher. However, because place of delivery occurs subsequently and may be related to both ANC source and mode of delivery, these findings should not be interpreted as evidence of a causal relationship. Rather, they indicate that the association between private ANC and CS is sensitive to how subsequent place of delivery is handled in the model.

Socioeconomic inequality was evident in all three settings. In Bangladesh, the pro-rich distribution of CS use was evident both in the regression analysis and in the concentration index, consistent with previous evidence from Bangladesh and other South Asian settings [22,45]. The mechanisms underlying this inequality may differ by context. In settings where CS prevalence is low, a pro-rich pattern may partly reflect underuse among poorer women who cannot access life-saving obstetric care. In Bangladesh, where overall CS use is already high, the same pro-rich pattern may reflect socioeconomic differences in access to and use of maternity services, care-seeking, and financial capacity, rather than solely reflecting underuse among poorer women. However, the available data do not establish whether poorer women who needed CS were able to receive it.

The findings on maternal age and parity are also consistent with clinical and social patterns reported in previous studies [46,47]. Older maternal age was associated with a higher prevalence of CS, although the strength of this association varied across settings. The relatively narrower age-related differences observed in Bangladesh may reflect the broader normalisation of CS among younger women. Higher parity was associated with a lower prevalence of CS in all three settings, but the association was weaker in Bangladesh than in West Bengal or Nepal. This may indicate that even multiparous women in Bangladesh are increasingly exposed to a health-system environment where surgical delivery has become common. However, this finding should be interpreted cautiously because the DHS does not contain detailed clinical information related to delivery decisions.

These findings have several implications for maternity-care policy and practice. In Bangladesh, monitoring CS use by facility type and socioeconomic group may help identify facilities or population groups that warrant further assessment. Facility-level review using the Robson classification may help identify which groups of women contribute most to CS use and inform quality-improvement strategies [38,48]. Such approaches should be accompanied by efforts to maintain timely access to medically indicated CS, particularly for poorer women who may face barriers to emergency obstetric care. Across settings, interventions should also consider provider practices, labour monitoring, institutional incentives, and informed counselling [49]. ANC should be strengthened as a platform for balanced birth planning, including clear information on when CS is medically necessary and when vaginal birth remains safe.

This study has several strengths. It used recent population-based data from Bangladesh, Nepal and West Bengal, allowing comparison across geographically and culturally related settings. Restricting the analysis to each woman’s most recent live birth reduced recall bias and avoided multiple births from the same woman. The use of adjusted prevalence ratios instead of odds ratios also improved interpretability because CS was a common outcome. In addition, concentration index analysis provided an equity-focused perspective beyond average prevalence estimates. The sensitivity analysis further allowed assessment of the private-facility association under an alternative facility-level comparison.

Several limitations should also be considered. DHS data are cross-sectional, so causality cannot be established. The datasets also lack detailed information on clinical indications, timing and urgency of CS, previous CS, maternal request, referral status and facility readiness. The available DHS data also provide limited information on women’s autonomy and decision-making regarding mode of delivery, precluding assessment of whether CS was patient-preferred, provider-recommended, or medically indicated. As a result, medically indicated and potentially unnecessary CS could not be distinguished. Place of delivery is also a complex variable because women may choose or be referred to facilities because of complications. ANC was measured mainly by number and source of visits rather than quality or continuity of care. The primary population-level private-facility analysis included home births in the non-private reference category, whereas the sensitivity analysis restricted the comparison to facility births; these analyses therefore represent different estimands. Finally, because West Bengal was purposively selected for its geographic, historical, cultural, and socioeconomic relevance to Bangladesh, findings from West Bengal should not be generalised to India as a whole, where substantial subnational differences exist in demographic, socioeconomic, and health-system characteristics [31].

In conclusion, CS use in Bangladesh has reached a level that warrants substantial health-system attention. While increased institutional delivery is a positive development, the high proportion of caesarean deliveries is concerning. Across all three settings, delivery in private for-profit facilities was strongly associated with CS use, while CS use showed a pro-rich socioeconomic distribution. Bangladesh needs a balanced strategy that ensures access to life-saving CS for women who need it, while identifying and reviewing potentially unnecessary procedures through stronger informed counselling, clinical governance, provider accountability, and facility-level monitoring.

## Supplementary Material

Supplementary File.docx

STROBE Checklist.docx

## Data Availability

The de-identified data used in this study were obtained from the Bangladesh Demographic and Health Survey 2022, Nepal Demographic and Health Survey 2022, and India National Family Health Survey 2019–2021. Access to these datasets requires registration and approval from the Demographic and Health Surveys Program. Researchers may request access through the program website at https://dhsprogram.com/data. All data used in this study are de-identified.
