# Supplementary material for: Caesarean section use and inequalities in Bangladesh in regional context: comparisons with West Bengal and Nepal
Source: Glob Health Action. 2026 Oct 22;19(1):2750044. doi: 10.1080/16549716.2026.2750044 (PMC13700475; doi:10.1080/16549716.2026.2750044)
Supplement: Supplementary file 1 [file ZGHA_A_2750044_SM4607.docx]

**STROBE Checklist**

**Manuscript: Caesarean Section Use and Inequalities in Bangladesh in Regional Context: Comparisons with West Bengal and Nepal**

| **Section / topic** | **Item** | **STROBE recommendation** | **Where reported in the manuscript / comments** |
| --- | --- | --- | --- |
| **TITLE AND ABSTRACT** | | | |
| **Title and abstract** | 1(a) | Indicate the study's design with a commonly used term in the title or the abstract. | p. 2 |
| **Title and abstract** | 1(b) | Provide in the abstract an informative and balanced summary of what was done and what was found. | pp. 2-3 (Abstract) |
| **INTRODUCTION** | | | |
| **Background/rationale** | 2 | Explain the scientific background and rationale for the investigation being reported. | pp. 4-5 (Background). |
| **Objectives** | 3 | State specific objectives, including any prespecified hypotheses. | p. 6 (final paragraph of Background). No prespecified hypothesis is stated. |
| **METHODS** | | | |
| **Study design** | 4 | Present key elements of study design early in the paper. | pp. 4-6 (Methods: Data source and study population; Statistical analysis). The cross-sectional analysis design was mentioned. |
| **Setting** | 5 | Describe the setting, locations, and relevant dates, including periods of recruitment, exposure, follow-up, and data collection. | pp. 4-5 (Methods: Bangladesh 2022, Nepal 2022, and West Bengal 2019-2021; births within five years preceding each survey). |
| **Participants** | 6(a) | Give the eligibility criteria, and the sources and methods of selection of participants. | pp. 4-5 (Methods: Data source and study population). |
| **Variables** | 7 | Clearly define all outcomes, exposures, predictors, potential confounders, and effect modifiers. Give diagnostic criteria, if applicable. | p. 5 (Methods: Outcome variable; Explanatory variables). No diagnostic criteria were applicable. |
| **Data sources/measurement** | 8* | For each variable of interest, give sources of data and details of methods of assessment (measurement). Describe comparability of assessment methods if there is more than one group. | pp. 4-6 (Methods: harmonised Births Recode data, variable definitions, survey procedures, and analysis). Comparability is addressed through harmonisation and common definitions. |
| **Bias** | 9 | Describe any efforts to address potential sources of bias. | pp. 6-7 (restriction to the most recent live birth to reduce recall bias and correlated observations); pp. 17-18 (limitations and residual sources of bias). |
| **Study size** | 10 | Explain how the study size was arrived at. | pp. 6-7. The study used all eligible complete records in the selected surveys; final samples are reported, but the numbers excluded at each stage are not reported. |
| **Quantitative variables** | 11 | Explain how quantitative variables were handled in the analyses. If applicable, describe which groupings were chosen and why. | p. 7 (Methods: categories for age, parity, education, wealth, and antenatal care visits). The rationale for some cut-points is not stated. |
| **Statistical methods** | 12(a) | Describe all statistical methods, including those used to control for confounding. | p. 6 (Methods: weighted descriptive analyses, concentration index and curves, and multivariable modified Poisson regressions; covariate adjustment listed). |
| **Statistical methods** | 12(b) | Describe any methods used to examine subgroups and interactions. | p. 6 (separate setting-specific models for Bangladesh, West Bengal, and Nepal). No interaction analyses are reported. |
| **Statistical methods** | 12(c) | Explain how missing data were addressed. | p. 7 No missing data were present for the outcome or prespecified covariates; therefore, no records were excluded and complete-case analysis was used. |
| **Statistical methods** | 12(d) | If applicable, describe analytical methods taking account of sampling strategy. | p. 6 (sampling weights, rescaling/normalisation, primary sampling-unit clustering, and robust variance estimation). |
| **Statistical methods** | 12(e) | Describe any sensitivity analyses. | Sensitivity analysis conducted and reported in Method, Results, Discussion and Supplementary Figure 1. |
| **RESULTS** | | | |
| **Participants** | 13(a)* | Report numbers of individuals at each stage of the study, for example, potentially eligible, examined for eligibility, confirmed eligible, included, and analysed. | p. 7 gives final analytical samples (Bangladesh 5,064; West Bengal 4,894; Nepal 2,942). |
| **Participants** | 13(b)* | Give reasons for non-participation at each stage. | p. 7 states no records were excluded for missing outcome or covariate data |
| **Participants** | 13(c)* | Consider use of a flow diagram. | Not included. No rigorous participant selection method was applied. |
| **Descriptive data** | 14(a)* | Give characteristics of study participants and information on exposures and potential confounders. | Table 1. |
| **Descriptive data** | 14(b)* | Indicate the number of participants with missing data for each variable of interest. | The Methods state that complete-case analysis was used (p. 7) |
| **Outcome data** | 15* | Report numbers of outcome events or summary measures. | Figure 1 (weighted caesarean-section prevalence overall and by facility type). |
| **Main results** | 16(a) | Give unadjusted estimates and, if applicable, confounder-adjusted estimates and their precision. Make clear which confounders were adjusted for and why they were included. | pp. 8-12, Figures 1-3, S1. |
| **Main results** | 16(b) | Report category boundaries when continuous variables were categorised. | pp. 7-8, Table 1. |
| **Main results** | 16(c) | If relevant, consider translating estimates of relative risk into absolute risk for a meaningful time period. | Not applicable. |
| **Other analyses** | 17 | Report other analyses done—for example, subgroup analyses, interactions, and sensitivity analyses. | pp. 8 and 10-12: setting-specific models and concentration-index analyses are reported. Sensitivity analyses are reported on p. 11. |
| **DISCUSSION** | | | |
| **Key results** | 18 | Summarise key results with reference to study objectives. | pp. 12-17 (Discussion) and p. 18 (Conclusion). |
| **Limitations** | 19 | Discuss limitations of the study, taking into account sources of potential bias or imprecision. Discuss both direction and magnitude of any potential bias. | pp. 17-18. Major limitations are described. |
| **Interpretation** | 20 | Give a cautious overall interpretation of results considering objectives, limitations, multiplicity of analyses, results from similar studies, and other relevant evidence. | pp. 12-18 (Discussion and Conclusion). |
| **Generalisability** | 21 | Discuss the generalisability (external validity) of the study results. | pp. 16-17 notes nationally representative data and related settings, and p. 17 discuss limitations. |
| **OTHER INFORMATION** | | | |
| **Funding** | 22 | Give the source of funding and the role of the funders for the present study and, if applicable, for the original study on which the present article is based. | p. 18 (Funding and Acknowledgement). |

** Give information separately for exposed and unexposed groups, where applicable.*

Source: STROBE Statement—Checklist of items that should be included in reports of cross-sectional studies. The checklist should be used with the STROBE Explanation and Elaboration document.
