# Supplementary material for: Caesarean section use and inequalities in Bangladesh in regional context: comparisons with West Bengal and Nepal
Source: Glob Health Action. 2026 Oct 22;19(1):2750044. doi: 10.1080/16549716.2026.2750044 (PMC13700475; doi:10.1080/16549716.2026.2750044)
Supplement: Supplementary file 2 [file ZGHA_A_2750044_SM4608.docx]

Supplementary Figure S1: Adjusted prevalence ratios for caesarean section associated with private-for-profit facility delivery among facility births in Bangladesh, West Bengal (India), and Nepal

Note: The analysis was restricted to women who delivered in a health facility, and private-for-profit facility delivery was compared with public not-for-profit facility delivery. The facility-birth sensitivity analysis included 3,266 women in Bangladesh, 4,532 in West Bengal, and 2,359 in Nepal. Forest plots show adjusted prevalence ratios (APRs) and 95% confidence intervals (CIs) from multivariable modified Poisson regression models with a log link. Models were adjusted for maternal age, parity, maternal education, household wealth quintile, place of residence, pregnancy intention, antenatal care utilization, receipt of antenatal care from private providers, and child sex, and accounted for the complex DHS survey design using sampling weights and clustering.
